# Tuning the Piezoelectric Performance of K_0.5_Na_0.5_NbO_3_ through Li Doping: Insights from Structural, Elastic and Electronic Analyses

**DOI:** 10.3390/ma17092118

**Published:** 2024-04-30

**Authors:** Hui Li, Tianxiang Zhou, Kang Xu, Han Wang, Wenke Lu, Jinyi Liu

**Affiliations:** School of Materials Science and Engineering, Chang’an University, Xi’an 710064, China; zhou_tx@chd.edu.cn (T.Z.); xk11252024@163.com (K.X.); 2023131021@chd.edu.cn (H.W.); luwenke420@gmail.com (W.L.); liujinyi_99@163.com (J.L.)

**Keywords:** Li-doped K_0.5_Na_0.5_NbO_3_, elastic properties, electronic properties, piezoelectric properties, first-principles

## Abstract

The structural, elastic, piezoelectric, and electronic properties of Li-doped K_0.5_Na_0.5_NbO_3_ (K_0.5−*x*_Na_0.5−*y*_Li*_x_*_+_*_y_*NbO_3_, KNN-L) are calculated. The properties of KNN-L are related to the Li-doping content and the replaced K or Na atoms. The bulk modulus, the shear modulus, and Young’s modulus of KNN-L are mostly higher than those of KNN, and the hardness value increases. The Poisson ratio of KNN-L is lower than that of most KNN, and the ductility is reduced. All doped structures are direct band gap semiconductors. K_0.5_Na_0.375_Li_0.125_NbO_3_ has the largest piezoelectric charge constant, *d*_33_ = 44.72 pC/N, in the respective structures, which is 1.5 fold that of K_0.5_Na_0.5_NbO_3_ (29.15 pC/N). The excellent piezoelectric performance of Li-doping KNN-L was analyzed from the insights of elastic and electronic properties.

## 1. Introduction

Piezoelectric materials can convert mechanical energy and electrical energy into each other and are widely used in the pressure sensor, piezoelectric memory, and photovoltaic fields [1]. Piezoelectric materials have important applications in daily life technology, and the national defense industry [2].

Piezoelectric ceramic materials are mainly divided into lead and lead-free piezoelectric ceramic materials. Pb(Zr*_x_*Ti_1−*x*_)O_3_, (1 − *x*)Pb(Mg_1/3_Nb_2/3_)O_3_−*x*PbTiO_3_ and other lead-containing piezoelectric materials have excellent elastic, dielectric, piezoelectric, pyroelectric, ferroelectric, electromechanical coupling and optical properties [3,4]. Therefore, they have an extremely important application value in the ultrasonic transducer, sensor, driver, filter, and memory fields. Although lead-containing piezoelectric materials are widely used because of their rich types, excellent performance and low cost [5], there is a big problem in the use of PZT-based ceramics, that is, the content of lead in its composition is more than 60%. The lead element is volatile and is a toxic substance, which brings a series of hazards to the environment and the human body in the process of use and recycling [6].

To develop new lead-free piezoelectric materials to replace PZT-based ceramics, researchers have conducted a lot of work in recent decades. At present, lead-free piezoelectric ceramic materials can be divided into five main categories: bismuth-containing layered structural materials, tungsten bronze structural materials, barium titanate (BaTiO_3_, BT)-based piezoelectric materials, bismuth-sodium titanate (Bi_0.5_Na_0.5_TiO_3_, BNT))-based piezoelectric materials and potassium sodium niobate (K_1−*x*_Na*_x_*NbO_3_, KNN)-based piezoelectric materials [7]. Among them, BT, BNT, and KNN-based lead-free piezoelectric materials are perovskite structures. Due to their morphotropic phase boundary (MPB) or polycrystalline phase boundary (PPB) structures, they exhibit excellent piezoelectric properties near the phase boundary components and have been widely studied. KNN-based ceramics have a higher Curie temperature and a lower sintering temperature than BT-based ceramics, and a higher piezoelectric constant than BNT-based ceramics, so they become the main piezoelectric materials that can replace PZT [8,9].

KNN-based ceramics have good application prospects, but their piezoelectric properties are still far inferior to PZT. At present, doping Li^+^ and other monovalent cations is the simplest and most efficient way to improve the piezoelectric properties [10]. Guo et al. [11] firstly reported the effect of A-position Li doping on the phase transition behavior and dielectric piezoelectric properties of KNN ceramics. They found the improved piezoelectric properties of *d*_33_ = 200~235 pC/N when 5~7% Li was added. Song et al. [12] studied the microstructure and piezoelectric properties of KNN ceramics doped with LiNbO_3_ (LN), and found the best piezoelectric constant *d*_33_ = 240 pC/N when the doping content of LN was 7%. The (K_0.5_Na_0.5_)NbO_3_-*x*LiNbO_3_ piezoelectric ceramics synthesized by Du et al. [13] showed an optimal value of *d*_33_ = 215 pC/N when the amount of LiNbO_3_ was 0.06 mol. In addition, the (1 − *x*)K_0.49_Na_0.51_NbO_3_−*x*LiNbO_3_ lead-free piezoelectric ceramics prepared by Shen et al. [14] also showed excellent electrical properties *d*_33_ = 246 pC/N at *x* = 0.06. It was found that the doping of Li can improve the piezoelectric properties of KNN. However, it is difficult to know experimentally that Li will replace K, Na or both K and Na in the A position.

For piezoelectric materials, the excellent piezoelectric properties are mainly due to the intrinsic contribution of crystal structure (polyphase coexistence and lattice distortion) and the extrinsic contribution of microstructure (grain size and domain structure). At present, the experimental research can only explore the extrinsic contribution of its microstructure, but cannot explore the intrinsic contribution of crystal structure in more detail [15]. Therefore, this paper will study the effect of crystal structure on piezoelectric properties of materials by first-principles method. The first-principles calculation method using density functional theory (DFT) and density functional perturbation theory (DFPT) has been able to effectively support the exploration of piezoelectric properties in different crystal structures [16,17,18]. In our previous report, it has been shown that LiTaO_3_ [19] and Li [20] can improve the piezoelectric performance of KNbO_3_. So, in this paper, we will further theoretically discuss the modification of Li on the structural, elastic, piezoelectric, and electronic properties of K_0.5_Na_0.5_NbO_3_.

## 2. Calculation Methods

In this paper, the first-principles calculations were carried out by the Vienna ab initio simulation package (VASP 5.2), which is based on density functional theory and the plane-wave pseudopotential methods [21,22]. The generalized gradient approximation (GGA) and the Perdew–Burke–Ernzerhofer (PBE) exchange-correlation function were used [23,24]. The valence electron configurations considered in the calculation were K (3s^2^3p^6^4s^1^), Na (2s^2^2p^6^3s^1^), Li (1s^2^2s^1^), Nb (4p^6^5s^1^4d^4^), and O (2s^2^2p^4^), respectively. The Monkhorst–Pack k-point sampling was generated with a 5 × 6 × 9 grid for the Brillouin zone integration [25]. During the structural optimization and properties calculations, the plane wave cutoff energy of 550 eV and the energy convergence of 10^−7^ eV were chosen. The structural optimization is obtained until the Hellmann–Feynman forces acting on each atom is less than 0.005 eV/Å. For the Born effective charge and piezoelectric stress constants calculations, the density functional perturbation theory was used [26].

## 3. Results and Discussion

### 3.1. Structural Properties

**Figure 1:**
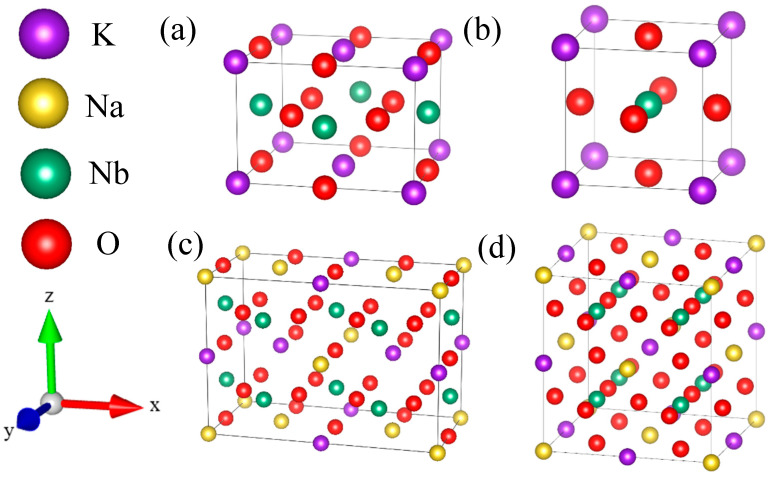
Schematic diagram of KNbO_3_ and K_0.5_Na_0.5_NbO_3_ crystal structure: (**a**) KNbO_3_ orthogonal phase unit structure, (**b**) KNbO_3_ tetragonal phase unit structure, (**c**) K_0.5_Na_0.5_NbO_3_ orthogonal phase structure, and (**d**) K_0.5_Na_0.5_NbO_3_ tetragonal phase structure.

The orthogonal (Bmm2) and tetragonal (P4mm) phases of the KNbO_3_ primitive cell are used as the basic structure in Figure 1a,b, and 50% of K is randomly replaced by Na to construct the 2 × 2 × 1 orthogonal and 2 × 2 × 2 tetragonal of the K_0.5_Na_0.5_NbO_3_ supercell in Figure 1c,d. They are used to study the influence of different phase structures on piezoelectric properties, and the optimized lattice parameters are shown in Table 1. The calculation results in Table 1 show that the calculated values in this paper are close to other calculated values and experimental values, and the error is less than 3%, which is within the allowable range of theory [27,28,29]. It can be considered that the first-principles method, related parameter settings and structural models adopted in this paper are accurate and reliable. Both the lattice parameters (*a*, *b*, *c*) and cell volume decrease when Na is doped. This is mainly because the atomic radiuses of Na (1.91 Å) are smaller than those of K (2.35 Å).

To explore the properties of Li-doped K_0.5_Na_0.5_NbO_3_ (K_0.5−*x*_Na_0.5−*y*_Li*_x_*_+_*_y_*NbO_3_, KNN-L) at room temperature, Na or K atoms were randomly replaced by Li atoms according to the orthorhombic K_0.5_Na_0.5_NbO_3_ in Figure 1c. Different Li concentrations *x* + *y* in K_0.5−*x*_Na_0.5−*y*_Li*_x_*_+*y*_NbO_3_ (*x* + *y* = 0.0625, 0.125, 0.25, 0.375, and 0.5) were modeled, and the optimized lattice parameters are also shown in Table 1. The results in Table 1 show that both the lattice parameters and cell volume decrease when Li is doped. The optimized lattice parameters when *x* + *y* = 0.0625 are consistent with the experiment results [33] of KNN-0.06Li and KNN-0.06Li. Moreover, the lattice parameters and cell volume of structures that K replaced by Li are smaller than those of structures that Na replaced by Li. This is mainly because the atomic radiuses of Li (1.57 Å) are smaller than those of K (2.35 Å) and Na (1.91 Å).

### 3.2. Formation Energy of Doped Systems

The formation energy is the energy required when the doped element enters the crystal structure to replace the replaced element. The smaller the value, the more easily the doped element will replace the original element, and the more stable the structure of the doped system will be. For all doped systems, the formation energy can be calculated using the following expressions [34,35]:

(1) $$E_{f}\left(K\to Li\right)=E\left(K_{0.5-x}{Na}_{0.5}{Li}_{x}NbO_{3}\right)-E\left(K_{0.5}{Na}_{0.5}NbO_{3}\right)+\mu_{K}-\mu_{Li}$$

(2) $$E_{f}\left(Na\to Li\right)=E\left(K_{0.5}{Na}_{0.5-y}{Li}_{y}NbO_{3}\right)-E\left(K_{0.5}{Na}_{0.5}NbO_{3}\right)+\mu_{Na}-\mu_{Li}$$

(3) $$E_{f}\left(K/Na\to Li\right)=E\left(K_{0.5-x}{Na}_{0.5-y}{Li}_{x+y}NbO_{3}\right)-E\left(K_{0.5}{Nb}_{0.5}O_{3}\right)+\mu_{K}+\mu_{Na}-\mu_{Li}$$

where $E_{f}$ is the formation energy; E is the substituted system energy of a supercell; μ is the chemical potential of each atom, and all the reference phases of K, Na, and Li are calculated using the structures with a Im-3m space group (No. 229).

The calculated formation energies of KNN-L are all listed in Table 1. As can be seen, the formation energies of all doped systems are negative, indicating that the structures of K_0.5_Na_0.5_NbO_3_ doped by Li atoms can exist stably. In addition, the formation energies of K_0.5−*x*_Na_0.5_Li*_x_*NbO_3_ are higher than those of K_0.5_Na_0.5−*y*_Li*_y_*NbO_3_. This means that the stability of the K_0.5_Na_0.5−*y*_Li*_y_*NbO_3_ structure is better, and it may be more inclined to form the K_0.5_Na_0.5−*y*_Li*_y_*NbO_3_ structure in practical applications.

### 3.3. Elastic Properties

The Born stability criteria are the basis for the determination of the stability of crystal mechanics. For crystal materials, when the elastic stiffness constants conform to the Born stability criteria, the structure can be identified as stable. For orthorhombic crystal structures, the elastic stiffness matrix has nine effective values (*C*_11_, *C*_12_, *C*_13_, *C*_22_, *C*_23_, *C*_33_, *C*_44_, *C*_55_, *C*_66_), and the Born stability criteria are given as follows [36]:

(4) $$C_{11}+C_{22}+C_{33}+2(C_{12}+C_{13}+C_{23})>0\;C_{11}+C_{22}>C_{12}\;C_{22}+C_{33}>{2C}_{23}\;C_{11}+C_{33}>{2C}_{13}\;C_{ij}>0\left(i,j=1,2,3,4,5,6\right)$$

The elastic constants *C*_ij_ of K_0.5−*x*_Na_0.5−*y*_Li*_x_*_+_*_y_*NbO_3_ were calculated and the results are shown in Table 2. It is shown that the elastic stiffness constants *C*_ij_ of all doped structures meet the Born stability criteria, that is, it can be assumed that all the doped structures with Li atoms are mechanically stable.

According to the calculated elastic stiffness matrix *C*_ij_, the bulk modulus (*B*), the shear modulus (*G*), Young’s modulus (*E*), Poisson’s ratio (*v*), and hardness (*G*/*B*) of all the doped systems can be calculated by the Voigt–Reuss–Hill approximation method. The upper (*B*_V_, *G*_V_) and lower (*B*_R_, *G*_R_) limits of the bulk modulus and the shear modulus, as well as the calculations of Young’s modulus (*E*), Poisson’s ratio (*v*), and hardness (*G*/*B*) are given by Formulas (5)–(10) for the ortho-phase crystal structure [37,38]. The calculation results of the elastic modulus and Poisson’s ratios are shown in Table 3.

(5) $$B_{V}=\left[\left(C_{11}+C_{22}+C_{33}\right)+2\left(C_{12}+C_{13}+C_{23}\right)\right]/9$$

(6) $$G_{V}=\left[\left(C_{11}+C_{22}+C_{33}-C_{12}-C_{13}-C_{23}\right)+3\left(C_{44}+C_{55}+C_{66}\right)\right]/15$$

(7) $$B_{R}=1/\left[\left(S_{11}{+S}_{22}{+S}_{33}\right)+2\left(S_{12}{+S}_{13}{+S}_{23}\right)\right]$$

(8) $$G_{R}=15/\left[4\left(S_{11}+S_{22}+S_{33}-S_{12}-S_{13}-S_{23}\right)+3\left(S_{44}+S_{55}+S_{66}\right)\right]$$

(9) E=(9G×B)/(3B+G)

(10) ν=(3B−2G)/[2(3×B+G)]

As can be seen from Table 3, the bulk modulus, the shear modulus, and Young’s modulus of KNN-L are mostly higher than those of KNN, and the hardness value increases. The Poisson ratio of KNN-L is lower than that of most KNN, and the ductility is reduced. The *B*_H_ of K_0.5−*x*_Na_0.5_Li*_x_*NbO_3_ is greater than that of K_0.5_Na_0.5−*y*_Li*_y_*NbO_3_ except for *x* = 0.125. When Li is doped in a small amount (*x* = 0.0625), the *v* of K_0.5−*x*_Na_0.5_Li*_x_*NbO_3_ is greater than that of K_0.5_Na_0.5−*y*_Li*_y_*NbO_3_, and the *G*_H_, *E* and *G*/*B* of K_0.5−*x*_Na_0.5_Li*_x_*NbO_3_ are smaller than the those of K_0.5_Na_0.5−*y*_Li*_y_*NbO_3_. However, when the doping amount of Li is increased (*x* = 0.125, 0.25, 0.375, 0.5), the *v* of K_0.5−*x*_Na_0.5_Li*_x_*NbO_3_ is less than that of K_0.5_Na_0.5−*y*_Li*_y_*NbO_3_, and the *G*_H_, *E* and *G*/*B* of K_0.5−*x*_Na_0.5_Li*_x_*NbO_3_ are greater than those of K_0.5_Na_0.5−*y*_Li*_y_*NbO_3_. This means that K_0.5−*x*_Na_0.5_Li*_x_*NbO_3_ is harder and less ductile than K_0.5_Na_0.5−*y*_Li*_y_*NbO_3_.

**Figure 2:**
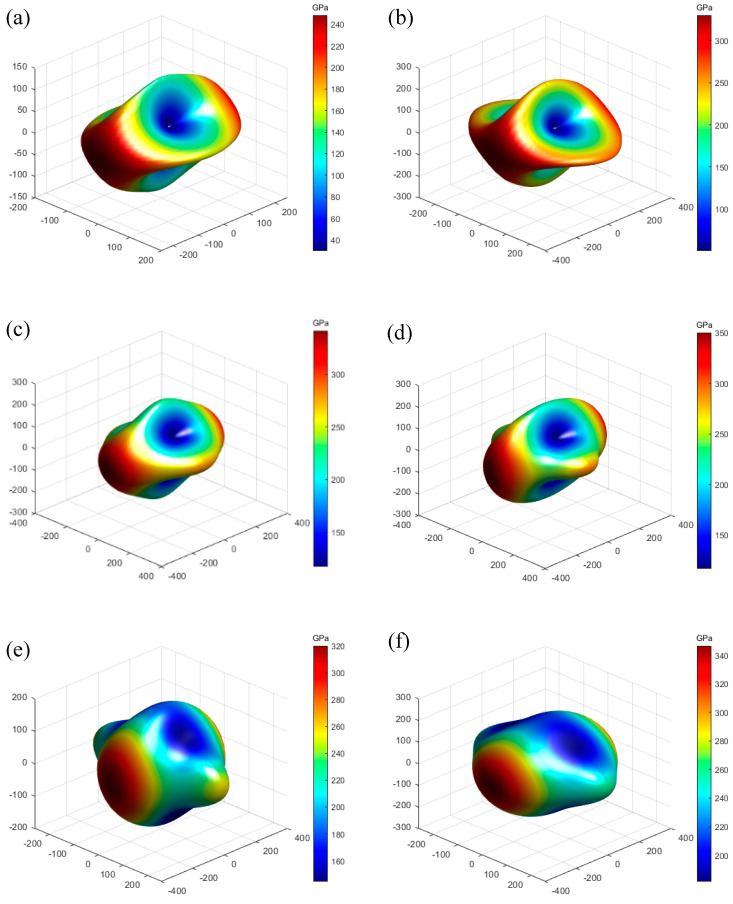
Three-dimensional curves of Young’s modulus *E* (in GPa) for Li doping K in K_0.5 −_*_x_*Na_0.5_Li*_x_*NbO_3_: (**a**) *x* = 0, (**b**) *x* = 0.0625, (**c**) *x* = 0.125, (**d**) *x* = 0.25, (**e**) *x* = 0.375, and (**f**) *x* = 0.5.

**Figure 3:**
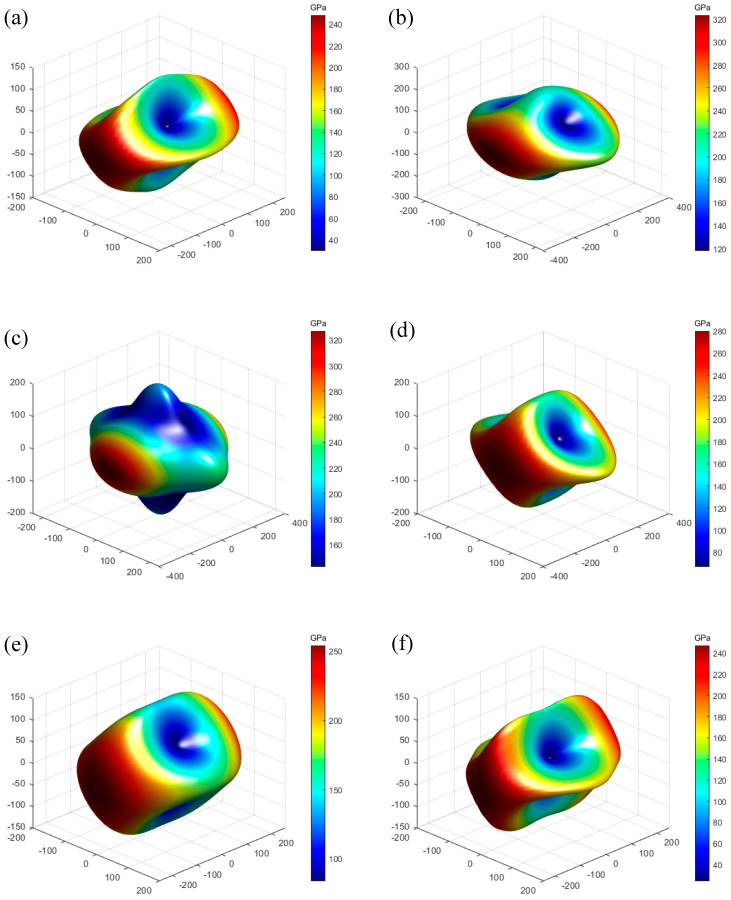
Three-dimensional curves of Young’s modulus *E* (in GPa) for Li doping Na in K_0.5_Na_0.5−*y*_Li*_y_*NbO_3_: (**a**) *y* = 0, (**b**) *y* = 0.0625, (**c**) *y* = 0.125, (**d**) *y* = 0.25, (**e**) *y* = 0.375, and (**f**) *y* = 0.5.

The three-dimensional curves of Young’s modulus are drawn according to the elastic modulus data, as shown in Figure 2 and Figure 3. The greater the difference between the 3D curves and the 3D sphere, the stronger the anisotropy of the material. Comparing K_0.5_Na_0.5_NbO_3_ in Figure 2a, it can be observed from Figure 2b–f that the 3D curves of Young’s modulus of the K_0.5−*x*_Na_0.5_Li*_x_*NbO_3_ structure are significantly less deviated from the sphere, indicating that the incorporation of Li atoms makes K_0.5−*x*_Na_0.5_Li*_x_*NbO_3_ less anisotropic.

In the K_0.5_Na_0.5−_*_y_*Li*_y_*NbO_3_ system, comparing K_0.5_Na_0.5_NbO_3_ in Figure 3a, the anisotropy of Young’s modulus of Figure 3c (*y* = 0.125) and Figure 3f (*y* = 0.5) increase, while the anisotropy of doped structures in Figure 3b,d,e decrease.

### 3.4. Piezoelectric Properties

In the perovskite material system, there is a linear coupling relationship between mechanical properties and electrical properties. The piezoelectric constant describing the linear relationship between force and electricity calculation formula is as follows [39]:

(11) $$d_{\alpha j}=\sum_{i=1}^{6}e_{\alpha i}S_{ij}$$

where *d*_αj_ refers to the piezoelectric charge tensor, α = 1–3 and j = 1–6; *e*_αi_ is the piezoelectric stress tensor, i = 1–6; and *S*_ĳ_ is the elastic compliance coefficient. Since *d*_αj_ and *e*_αi_ are 3 × 6 matrices and *S*_ĳ_ is 6 × 6 matrices, we use α, i, and j to distinguish the rows and columns. In the piezoelectric charge tensor *d*_αj_, *d*_33_ is the most commonly used piezoelectric constant to characterize the performance of a piezoelectric material.

**Figure 4:**
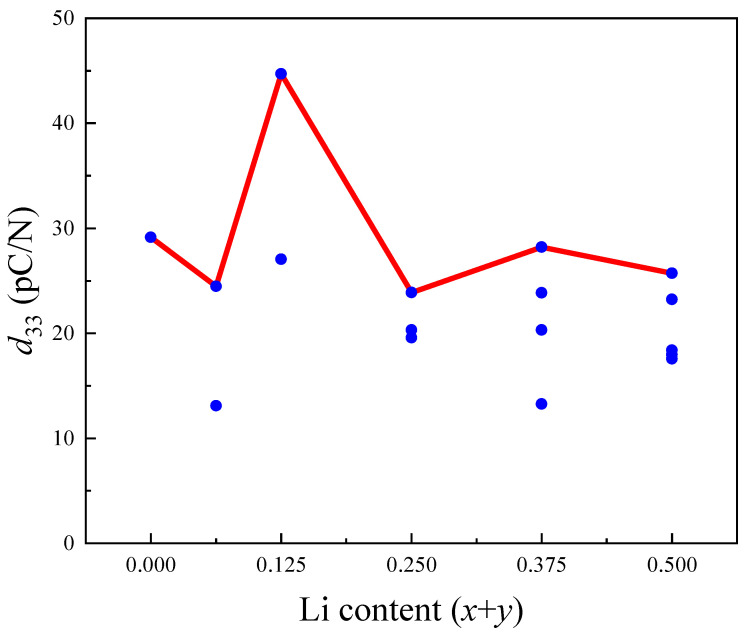
The piezoelectric charge tensor *d*_33_ of KNN-L with different Li content.

The *e*_33_ and *d*_33_ of KNbO_3_ and K_0.5−*x*_Na_0.5−*y*_Li*_x_*_+_*_y_*NbO_3_ are also shown in Table 1. The *d*_33_ of KNbO_3_ and K_0.5_Na_0.5_NbO_3_ tetragonal phase are higher than that of KNbO_3_ and K_0.5_Na_0.5_NbO_3_ orthogonal phase. It can be inferred that the excellent piezoelectric properties in the polyphase coexisting region, explained from the aspect of crystal structure, may be due to the tetragonal phase with good piezoelectric properties compensating for the orthogonal phase with poor piezoelectric properties. In addition, the *d*_33_ of K_0.5−*x*_Na_0.5−*y*_Li*_x_*_+_*_y_*NbO_3_ reached the maximum value (44.72 pC/N) when *x* = 0.125, and compared with the *d*_33_ of K_0.5_Na_0.5_NbO_3_ (29.15 pC/N), it is 1.5-fold higher. In order to more clearly reflect the variation trend of *d*_33_ with Li content, Figure 4 was plotted with all the *d*_33_ values (blue dots) of K_0.5−*x*_Na_0.5−*y*_Li*_x_*_+_*_y_*NbO_3_ in Table 1. As shown in Figure 4, the maximum *d*_33_ (solid red line) for each Li content first increased and then decreased with the increase in Li, which is consistent with the experimental results.

### 3.5. The Born Effective Charge

In piezoelectric materials with perovskite structure, a larger Born effective charge tends to generate a larger spontaneous field. For piezoelectric materials, the greater the spontaneous polarization, the better the piezoelectric characteristics. Usually, the Born effective charge (Z*) can be obtained by using the following equation [40]:

(12) $$P_{\alpha }=\frac{e}{\Omega }\sum Z_{i\alpha \beta }^{*}\delta_{ui\beta }$$

where $P_{\alpha }$ means the macroscopic polarization; i,α,β represent the *i*-th atom, the direction of the polarization component, and atomic displacement, respectively;$\;\delta_{u}$ represents the atomic displacement.

To investigate the effect of Li doped on piezoelectric properties, the Born effective charges of KNbO_3_, K_0.5_Na_0.5_NbO_3_ and K_0.5_Na_0.375_Li_0.125_NbO_3_ are calculated by the DFPT method, and the results are shown in Table 4. As can be seen from Table 4, due to the addition of Li, the $\bar{Z*}$ of Nb and O_I_ atoms in K_0.5_Na_0.375_Li_0.125_NbO_3_ structure increase compared with those in KNbO_3_ and K_0.5_Na_0.5_NbO_3_. This is also consistent with the calculated results of piezoelectric properties.

### 3.6. Band Structure

**Figure 5:**
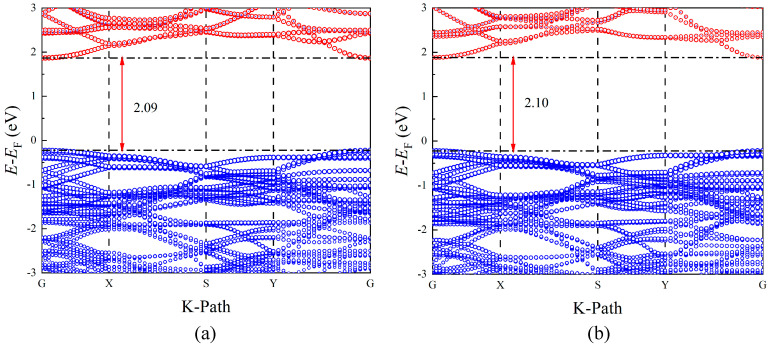
Band structures of undoped and Li-doped KNN: (**a**) K_0.5_Na_0.5_NbO_3_ and (**b**) K_0.5_Na_0.375_Li_0.125_NbO_3_. (The red line is Nb contributing band, and the blue line is O contributing band).

The band structures are shown in Figure 5. In order to calculate the band, the high-symmetry point path of G-X-S-Y-G is used. As can be seen from Figure 5, K_0.5_Na_0.5_NbO_3_ and K_0.5_Na_0.375_Li_0.125_NbO_3_ are all the direct band gap semiconductors, and the valence band maximum (VBM) and the conduction band minimum (CBM) are all located at the highly symmetric G-point. A direct band gap semiconductor has better energy utilization and electrical performance because its electrons will directly transition to a conduction band from a valence band when excited. In addition, the calculations show that the direct band gap of 2.09 eV for K_0.5_Na_0.5_NbO_3_ (comparable to the calculated result of 2.21 eV [31] and experiment value of 3.11 eV [41]) and 2.10 eV for K_0.5_Na_0.375_Li_0.125_NbO_3_ (1.96 eV [31]) is not much different. Li makes the CBM (1.87 to 1.88 eV) of K_0.5_Na_0.375_Li_0.125_NbO_3_ slightly move towards the higher energy level, and VBM (−0.22 eV) of K_0.5_Na_0.375_Li_0.125_NbO_3_ is consistent with K_0.5_Na_0.5_NbO_3_.

### 3.7. Density of States

**Figure 6:**
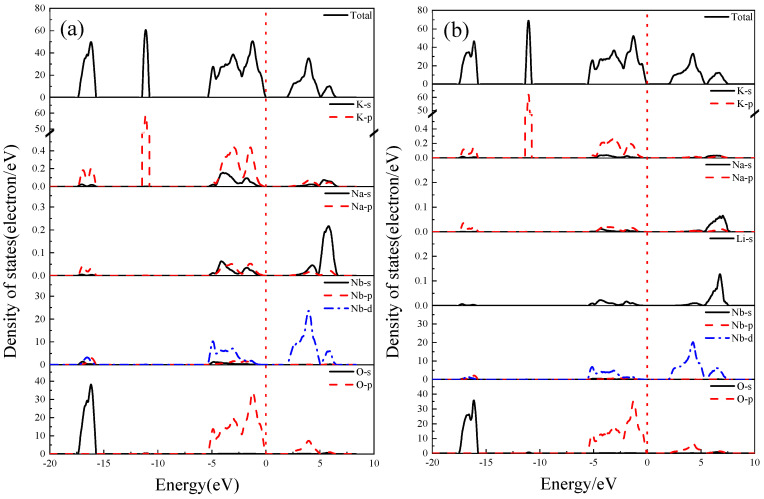
DOS and PDOS of undoped and Li-doped KNN: (**a**) K_0.5_Na_0.5_NbO_3_ and (**b**) K_0.5_Na_0.375_Li_0.125_NbO_3_.

The total and partial density of states (DOS and PDOS) of K_0.5_Na_0.5_NbO_3_ and K_0.5_Na_0.375_Li_0.125_NbO_3_ are calculated, and the calculation results are shown in Figure 6. The DOS main peaks of K_0.5_Na_0.375_Li_0.125_NbO_3_ are mainly concentrated in four regions (−17~−15 eV, −12~−10 eV, −5~0 eV, 2~7 eV), which are similar to those of K_0.5_Na_0.5_NbO_3_.

For the K_0.5_Na_0.375_Li_0.125_NbO_3_ structure in Figure 6b, the valence band (−5~0 eV) near the Fermi surface is mainly composed of s and p orbitals of K, Na and Li, O-2p, and Nb-4d orbitals. Similar to K_0.5_Na_0.5_NbO_3_, the component of the conduction band is also contributed by O-2p and Nb-4d orbitals. However, compared with K_0.5_Na_0.5_NbO_3_, Li reduces the contribution of K and Na to the valence band and conduction band, making the peak shape sharp and the conduction band move towards a higher energy level. This indicates that the Li has a strong effect on the electronic structure of K_0.5_Na_0.5_NbO_3_.

## 4. Conclusions

In this paper, the structural, elastic, piezoelectric, and electronic properties of Li-doped K_0.5−*x*_Na_0.5−*y*_Li*_x_*_+_*_y_*NbO_3_ were calculated using VASP software. The influence of different proportions of KNN-L was explored. The results show that the properties of KNN-L are related to the Li-doping content and the replaced K or Na atoms. The stability of the Na-replaced K_0.5_Na_0.5−*y*_Li*_y_*NbO_3_ structure is better than that of the K-replaced K_0.5−*x*_Na_0.5_Li*_x_*NbO_3_, and it may be more inclined to form the K_0.5_Na_0.5−*y*_Li*_y_*NbO_3_ structure in experiments. The bulk modulus, the shear modulus and Young’s modulus of KNN-L are mostly higher than those of KNN, and the hardness value increases. The Poisson ratio of KNN-L is lower than that of most KNN, and the ductility is reduced. The incorporation of Li atoms makes K_0.5-*x*_Na_0.5_Li*_x_*NbO_3_ less anisotropic, but the anisotropy of K_0.5_Na_0.5−*y*_Li*_y_*NbO_3_ decreases first and then increases with the addition of Li atoms. All doped systems are direct band gap semiconductors. The band gaps of K_0.5_Na_0.5_NbO_3_ (2.09 eV) and K_0.5_Na_0.375_Li_0.125_NbO_3_ (2.10 eV) are similar. Among the Li-doped KNN-L structures, K_0.5_Na_0.375_Li_0.125_NbO_3_ has the largest piezoelectric charge constant, *d*_33_ = 44.72 pC/N, which is 1.5 fold that of K_0.5_Na_0.5_NbO_3_ (29.15 pC/N). The results show that the Li-doped K_0.5_Na_0.5_NbO_3_ system helps to improve its piezoelectric capacity.

## Figures and Tables

**Table 1:** Lattice parameters, formation energy, piezoelectric properties (*e*_33_ and *d*_33_) of KNbO_3_, K_0.5_Na_0.5_NbO_3_ and Li-doped K_0.5−*x*_Na_0.5−*y*_Li*_x_*_+*y*_NbO_3_.

	Structure		*a* (Å)	*b* (Å)	*c* (Å)	*V* (Å^3^)	*E_f_* (eV)	*e*_33_	*d_33_*
KNbO_3_	Orthogonal (Bmm2)	Present	5.842	4.017	5.886	138.1	/	3.00	23.74
		Calc [19]	5.832	4.020	5.869	/	/	3.02	24.5
		Calc [27]	5.789	3.991	5.822	134.5	/	/	23.25
		Expt [28]	5.697	3.971	5.721	129.4	/	/	/
	Tetragonal (P4mm)	Present	4.028	4.028	4.256	69.1	/	3.80	66.17
		Calc [27]	4.003	4.003	4.197	67.3	/	/	54.40
		Expt [29]	3.996	3.996	4.063	64.9	/	/	/
K_0.5_Na_0.5_NbO_3_	Orthogonal (Bmm2)	Present	5.764	3.992	5.838	134.3	/	3.96	29.15
		Expt. [30]	5.657	4	4	/	/	/	/
		Calc [27]	/	/	/	/	/	/	29.12
	Tetragonal (P4mm)	Present	4.003	4.003	4.198	67.3	/	4.81	69.44
		Calc. [31]	3.978	3.978	3.931	/	/	4.14	149
		Expt. [32]	/	/	/	/	/	/	80
K_0.5−*x*_Na_0.5−*y*_Li*_x_*_+*y*_NbO_3_	*x* + *y* = 0.06	Expt. [33]	5.622	3.943	5.670	125.702	/	/	187
	*x* + *y* = 0.07	Expt. [33]	5.621	3.935	5.662	125.270	/	/	/
	*x* + *y* = 0.0625	*x* = 0.0625/*y* = 0	5.659	3.937	5.700	127.0	−12.63	3.55	12.21
		*x* = 0/*y* = 0.0625	5.675	3.946	5.707	127.8	−12.97	5.29	24.49
	*x* + *y* = 0.125	*x* = 0.125/*y* = 0	5.635	3.916	5.691	125.6	−6.31	6.98	27.04
		*x* = 0/*y* = 0.125	5.684	3.949	5.708	128.1	−6.63	7.97	44.72
	*x* + *y* = 0.25	*x* = 0.25/*y* = 0	5.596	3.887	5.687	123.7	−6.42	5.10	20.33
		*x* = 0/*y* = 0.25	5.690	3.948	5.746	129.1	−6.85	3.72	19.58
		*x* = 0.125/*y* = 0.125	5.617	3.912	5.734	126.0	−6.57	4.31	23.89
	*x* + *y* = 0.375	*x* = 0.375/*y* = 0	5.592	3.839	5.651	121.3	−6.90	4.22	20.33
		*x* = 0/*y* = 0.375	5.701	3.962	5.765	130.2	−7.28	4.26	28,20
		*x* = 0.125/*y* = 0.25	5.613	3.899	5.786	126.6	−6.87	3.60	23.86
		*x* = 0.25/*y* = 0.125	5.605	3.898	5.696	124.5	−6.67	2.71	13.26
	*x* +*y* = 0.5	*x* = 0.5/*y* = 0	5.577	3.898	5.723	124.4	−5.75	4.19	17.97
		*x* = 0/*y* = 0.5	5.686	3.941	5.832	130.7	−6.98	3.38	25.72
		*x* = 0.125/*y* = 0.375	5.653	3.926	5.752	127.7	−6.64	3.64	23.23
		*x* = 0.375/*y* = 0.125	5.557	3.898	5.647	122.3	−6.94	2.63	18.39
		*x* = 0.25/*y* = 0.25	5.667	3.933	5.723	127.6	−6.77	3.48	17.56

**Table 2:** The calculated elastic constants *C*_ij_ (in GPa) of K_0.5−*x*_Na_0.5−*y*_Li*_x_*_+*y*_NbO_3_.

Li Content (*x* + *y*)	*x* and *y*	Elastic Stiffness Coefficients								
		*C*_11_	*C*_12_	*C*_13_	*C*_22_	*C*_23_	*C*_33_	*C*_44_	*C*_55_	*C*_66_
0	Present	189.4	84.5	42.0	313.0	80.6	152.6	56.7	8.2	71.8
	Calc [27]	189.5	80.5	33.1	327.7	72.9	149.2	66.8	12.2	77.1
0.0625	*x* = 0.0625/*y* = 0	313.3	104.6	94.7	386.7	105.0	278.8	100.5	13.6	108.0
	*x* = 0/*y* = 0.0625	275.4	111.3	85.1	391.8	105.6	244.7	85.2	37.3	95.3
0.125	*x* = 0.125/*y* = 0	307.3	78.5	71.1	376.2	76.5	249.8	91.8	36.1	103.4
	*x* = 0/*y* = 0.125	280.5	110.6	82.7	399.5	110.7	239.9	57.9	48.2	83.4
0.25	*x* = 0.25/*y* = 0	318.7	68.9	77.1	382.6	79.2	246.9	105.4	35.8	91.3
	*x* = 0/*y* = 0.25	240.9	91.9	79.9	347.1	107.2	211.5	80.0	19.4	87.3
	*x* = 0.125/*y* = 0.125	259.1	55.2	52.9	335.3	72.8	191.9	90.3	29.5	78.8
0.375	*x* = 0.375/*y* = 0	285.2	65.8	64.4	343.7	54.2	200.9	92.2	49.5	82.0
	*x* = 0/*y* = 0.375	201.5	85.7	53.1	316.3	85.9	170.7	73.3	26.9	77.2
	*x* = 0.125/*y* = 0.25	239.8	55.0	48.3	314.7	79.2	170.1	84.5	20.5	70.1
	*x* = 0.25/*y* = 0.125	233.5	79.2	37.3	282.4	65.6	164.4	87.0	12.2	74.5
0.5	*x* = 0.5/*y* = 0	329.9	121.0	126.0	413.4	116.9	272.4	100.1	61.6	95.5
	*x* = 0/*y* = 0.5	199.9	82.1	47.0	310.2	83.1	151.2	70.8	6.6	67.7
	*x* = 0.125/*y* = 0.375	208.5	34.5	51.1	225.6	49.2	168.3	74.2	12.5	71.6
	*x* = 0.375/*y* = 0.125	133.9	39.9	3.4	144.7	16.3	147.8	80.6	42.3	85.1
	*x* = 0.25/*y* = 0.25	232.1	101.7	83.9	317.9	84.6	201.8	73.8	11.1	81.8

**Table 3:** The calculated elastic modulus (in GPa) of K_0.5−*x*_Na_0.5−*y*_Li*_x_*_+*y*_NbO_3_.

Li Content (*x* + *y*)	*x* and *y*	*B*_V_	*B*_R_	*B*_H_	*G*_V_	*G*_R_	*G*_H_	*E*	*ν*	*G*/*B*
0	*x* = 0/*y* = 0	118.8	103.1	110.9	57.2	27.3	42.2	112.4	0.331	0.38
0.0625	*x* = 0.0625/*y* = 0	176.4	172.2	174.3	89.4	44.0	66.7	177.4	0.330	0.38
	*x* = 0/*y* = 0.0625	168.4	160.1	164.2	84.2	71.1	77.7	201.3	0.296	0.47
0.125	*x* = 0.125/*y* = 0	153.9	149.7	151.8	93.4	76.0	84.7	214.2	0.265	0.56
	*x* = 0/*y* = 0.125	169.8	159.8	164.8	79.0	63.0	71.0	186.2	0.312	0.43
0.25	*x* = 0.25/*y* = 0	155.4	151.5	153.4	94.7	76.0	85.3	216.0	0.265	0.56
	*x* = 0/*y* = 0.25	150.8	143.2	147.0	72.0	50.1	61.1	160.9	0.318	0.42
	*x* = 0.125/*y* = 0.125	127.6	121.1	124.3	80.1	63.2	71.6	180.3	0.258	0.58
0.375	*x* = 0.375/*y* = 0	133.2	127.1	130.1	87.8	79.3	83.6	206.5	0.236	0.64
	*x* = 0/*y* = 0.375	126.4	111.9	119.2	66.4	53.8	60.1	154.3	0.284	0.50
	*x* = 0.125/*y* = 0.25	121.1	113.3	117.2	71.2	50.4	60.8	155.5	0.279	0.52
	*x* = 0.25/*y* = 0.125	116.1	107.0	111.5	67.9	37.8	52.9	137.0	0.295	0.47
0.5	*x* = 0.5/*y* = 0	193.7	189.1	191.4	94.9	89.1	92.0	237.9	0.293	0.48
	*x* = 0/*y* = 0.5	120.6	106.2	113.4	59.0	23.8	41.4	110.6	0.337	0.36
	*x* = 0.125/*y* = 0.375	96.9	88.4	92.7	62.8	34.0	48.4	123.7	0.278	0.52
	*x* = 0.375/*y* = 0.125	60.6	59.1	59.9	66.0	60.7	63.4	140.5	0.109	1.06
	*x* = 0.25/*y* = 0.25	143.6	137.9	140.8	65.5	35.2	50.3	134.8	0.340	0.36

**Table 4:** The Born effective charge of KNbO_3_, K_0.5_Na_0.5_NbO_3_ and K_0.5_Na_0.375_Li_0.125_NbO_3_.

Materials	Species	$Z_{xx}^{*}$	$Z_{yy}^{*}$	$Z_{zz}^{*}$	$\bar{Z*}$
KN	K	1.208	1.135	1.177	1.173
	Nb	7.454	8.836	6.243	7.511
	O_I_	−1.354	−6.915	−1.437	−3.236
	O_II_	−3.654	−1.528	−2.992	−2.724
KNN	K	1.169	1.150	1.170	1.163
	Na	1.234	1.142	1.140	1.172
	Nb	7.664	8.740	6.473	7.625
	O_I_	−1.378	−7.068	−1.430	−3.292
	O_II_	−3.779	−1.469	−3.117	−2.788
KNN-L	K	1.179	1.076	1.152	1.136
	Na	1.342	1.324	1.320	1.329
	Li	1.067	1.056	0.993	1.039
	Nb	7.888	8.739	7.260	7.962
	O_I_	−1.513	−7.087	−1.504	−3.368
	O_II_	−3.444	−1.476	−3.234	−2.718

## Data Availability

Data are contained within this article.
